# Mitochondrial Dysfunction as a Hallmark of Environmental Injury

**DOI:** 10.3390/cells11010110

**Published:** 2021-12-30

**Authors:** Carolina Duarte-Hospital, Arnaud Tête, François Brial, Louise Benoit, Meriem Koual, Céline Tomkiewicz, Min Ji Kim, Etienne B. Blanc, Xavier Coumoul, Sylvie Bortoli

**Affiliations:** 1 Environmental Toxicity, Therapeutic Targets, Cellular Signaling and Biomarkers, T3S, INSERM UMR-S 1124, F-75006 Paris, France; carolina.duarte_hospital@etu.parisdescartes.fr (C.D.-H.); arnaud.tete@parisdescartes.fr (A.T.); francois.brial@crc.jussieu.fr (F.B.); louise.am.benoit@gmail.com (L.B.); meriem.koual@aphp.fr (M.K.); celine.tomkiewicz@inserm.fr (C.T.); min-ji.kim@inserm.fr (M.J.K.); etienne.blanc@u-paris.fr (E.B.B.); 2 Faculty of Sciences, Université de Paris, F-75006 Paris, France; 3 Université Sorbonne Paris Nord, F-93000 Bobigny, France

**Keywords:** mitotoxicity, xenobiotics, environmental pollutants

## Abstract

Environmental factors including diet, sedentary lifestyle and exposure to pollutants largely influence human health throughout life. Cellular and molecular events triggered by an exposure to environmental pollutants are extremely variable and depend on the age, the chronicity and the doses of exposure. Only a fraction of all relevant mechanisms involved in the onset and progression of pathologies in response to toxicants has probably been identified. Mitochondria are central hubs of metabolic and cell signaling responsible for a large variety of biochemical processes, including oxidative stress, metabolite production, energy transduction, hormone synthesis, and apoptosis. Growing evidence highlights mitochondrial dysfunction as a major hallmark of environmental insults. Here, we present mitochondria as crucial organelles for healthy metabolic homeostasis and whose dysfunction induces critical adverse effects. Then, we review the multiple mechanisms of action of pollutants causing mitochondrial toxicity in link with chronic diseases. We propose the Aryl hydrocarbon Receptor (AhR) as a model of “exposome receptor”, whose activation by environmental pollutants leads to various toxic events through mitochondrial dysfunction. Finally, we provide some remarks related to mitotoxicity and risk assessment.

## 1. Introduction

Age-related diseases such as metabolic syndrome, multiple cancers and neurological pathologies are strongly related to mitochondrial dysfunctions. Mitochondrion is a cellular organelle that originates from the endosymbiosis of bacteria (Rickettsies), about 2 billion years ago as argued by Lynn Margulis in 1966, and then supported by the discovery of mitochondria-specific DNA (mtDNA that is inherited uniparentally solely from the mother) in 1980. The original DNA of the bacterium seems to have undergone various evolutions, losing a large number of genes, sometimes transferred into the genomic DNA of the host. A mitochondrion can only derive from another mitochondrion. The number of mitochondria per cell is regulated by cell activity and metabolic consumption. For example, the number of mitochondria in a muscle cell can vary according to its activity (resting muscle vs. trained muscle) from 5 to 10 times.

The central function of mitochondria is to provide the majority of cellular energy in the form of adenosine triphosphate (ATP), mainly through the oxidation of carbohydrates and fatty acids and the coupling of the Krebs cycle with the electron transfer chain, also known as the mitochondrial respiratory chain (MRC). The metabolites generated by these reactions are used for the biosynthesis of macromolecules such as lipids, proteins and nucleic acids. Mitochondria also play major roles in multiple cellular processes, including calcium (Ca^2+^) homeostasis, production of reactive oxygen species (ROS), cell death and production of second messengers.

Multiple natural products target the mitochondria. Yue Yang et al., in 2020, have listed these molecules as diverse as terpenoids (lupeol, betulinic acid), flavonoids (including flavones, isoflavones, flavanols, flavonols, and anthocyanidins), saponins (gracillin, polyphyllin I), alkaloids (codeine, morphine), coumarins (dentatin), quinones (plumbagin) or ichtyotoxins (rotenone). Various molecular processes are impacted by these natural substances, including generation of ROS, eventually leading to dysfunctions as decreased respiration and therefore ATP production, mitochondrial hyperfission, release of cytochrome *c* and subsequently activation of caspases and apoptosis [1]. Most of these molecules may have transient effects because they are metabolized by the cells and therefore deactivated.

**Figure 1:**
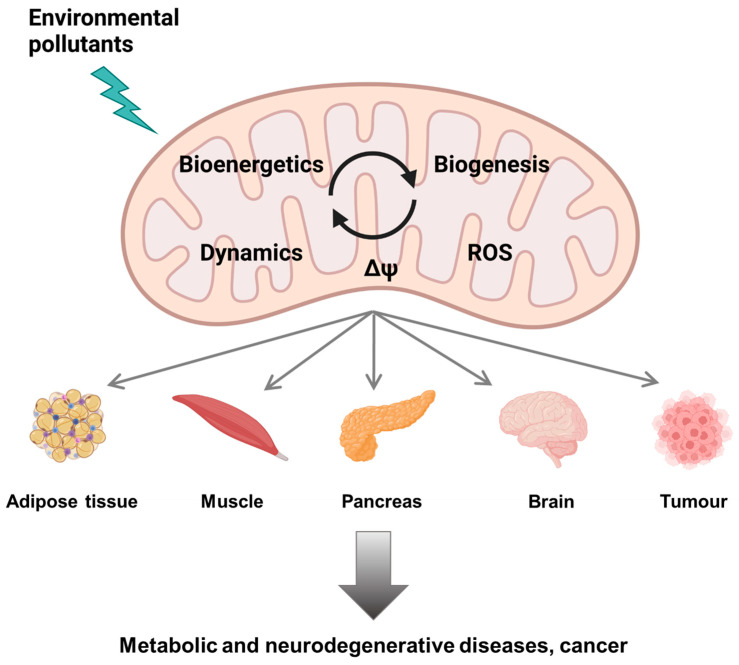
Mitochondrial dysfunction by environmental pollutants (created with BioRender.com, accessed on 30 November 2021).

Xenobiotics such as pollutants and drugs also target multiple macromolecules (DNA, proteins, lipids), including components of the mitochondria suggesting that their mechanisms of toxicity could also be related to mitochondrial deregulation (Figure 1). Recent advances in mechanistic toxicology confirm this statement and provide some tracks on the involvement of mitochondrial impairment in the toxicity of environmental contaminants.

## 2. Mitochondria as Crucial Organelles: From Healthy Metabolic Homeostasis to Critical Dysfunction

Mitochondria are eukaryotic cellular organelles at the origin of energy production, mainly ATP, necessary for the cellular function and metabolic homeostasis. For example, 50% of ATP is consumed for the translation of proteins. The number of mitochondria varies according to the cell type [2]: some cells contain only about 10 or even one mitochondrion while others may contain more than 100,000 of these organelles. In somatic cells of mammals, this number typically comprises between ~80 to ~2000. By contrast, Xenopus laevis oocytes appear to contain about 10^7^ mitochondria [2]. Their size (area between 0.75 and 3 μm^2^/mitochondrion) and shape are also closely related to cellular activity, including energy production, metabolite turnover and redox signaling [3]. Mitochondria form a dynamic network finely regulated by fission and fusion leading to granular and filamentous forms, respectively [4]. This network surrounds the cell nucleus and is associated with the microtubules of the cytoskeleton as well as with other cellular organelles. This proximity allows the ATP needs of each cellular process to be properly supplied.

The crucial importance of maintaining the integrity of mitochondrial functions for health is illustrated by the existence of numerous mitochondrial diseases. Some mitochondriopathies are genetic disorders involving mutations in mtDNA [5], such as Pearson syndrome, Addison’s disease, LHON (Leber hereditary optic neuropathy), MELAS (mitochondrial myopathy, encephalopathy, lactic acidosis and stroke-like episodes syndrome), MERRF (myoclonic epilepsy with ragged red fibers), NARP (neurogenic muscle weakness, ataxia and retinitis pigmentosa), MNGIE (mitochondrial neurogastrointestinal encephalopathy) syndrome and familial bilateral striatal necrosis [5,6]. They often present a wide range of clinical symptoms with a large interindividual variability, making extremely complex both their diagnosis and treatment. Some mitochondrial diseases result in the dysfunction of a single tissue, while others affect multiple tissues. For the same mutation, the pattern of disease expression as well as the age at which the first clinical symptoms occur can vary from one patient to another [6,7]. The clinical heterogeneity in patients with mitochondriopathies caused by mutations in mtDNA could be linked to heteroplasmy, i.e., in an individual cell, the presence of mitochondria bearing a mixture of mutated and normal mtDNA in varying proportions [6]. Other mitochondrial diseases involve mutations in nuclear genes encoding proteins implicated in mtDNA and dynamic maintenance such as Parkinson’s disease [8], Charcot-Marie-Tooth disease [9], dominant optic atrophy [10] and amyotrophic lateral sclerosis (ALS) [11].

The mitochondrion is a complex organelle, delimited as two compartments by an outer and an inner membrane: the intermembrane space and the matrix. Invaginations of the inner membrane form cristae that extend into the matrix. Thus, the number of mitochondria, or rather the volume of the mitochondrial network at steady state, is the result of the balance between biogenesis and degradation. One mitochondrion contains 2 to 10 copies of an extrachromosomal genome (mtDNA) composed by a histone-free double strand circular DNA molecule. mtDNA comprises 37 genes that encoding 22 tRNAs, 12S and 16S rRNA subunits and 13 polypeptides which, together with the 74 polypeptides encoded by nuclear DNA, constitute the enzymatic complexes of the electron transport system. mtDNA has its own replication, transcription and translation system. Its mutation rate is 10 to 17 times higher than that of nuclear DNA [5] since (i) it is not protected by histones, (ii) it is close to areas of ROS production, and (iii) mitochondria lack the efficient DNA repair mechanisms found in the nucleus.

Historically, mitochondria are known to be the powerhouse of eukaryotic cells, according to tight interconnections between the cytoplasmic glycolysis, the mitochondrial tricarboxylic acid cycle (TCA or Krebs cycle) and the respiratory chain. Thus, the matrix represents the most active area for ATP synthesis, the final step of the mitochondrial respiration. The remarkable plasticity of mitochondrial metabolism is characterized by a constant adjustment of the cellular mitochondrial content to adapt the energy production capacity to physiological or pathological demands. Besides its key role as a cell powerhouse through oxidative phosphorylation, mitochondria are a crucial hub of various metabolic pathways including Krebs cycle, urea cycle, fatty acid oxidation, gluconeogenesis and ketogenesis [12]. Disruption of metabolic homeostasis is critical in a number of diseases including neuropathies, metabolic diseases and cancers. A metabolic reprogramming occurs during neuronal development and appears to be a regulatory mechanism to drive the fate of neural stem cells (NSCs). During embryonic and adult neurogenesis, neuronal differentiation of NSCs requires a metabolism shift from a predominantly glycolytic metabolism to an oxidative metabolism based on efficient oxidative phosphorylation (OXPHOS) [13]. Mounting evidence suggests that impaired mitochondrial respiration might compromise neurogenesis. In various neuropathies and neurodegenerative diseases, decreased electron complex transfer activity and reduced ATP production are frequently observed [13]. Clinical and in vivo studies have highlighted that insulin resistance occurring in obesity and type 2 diabetes (T2D) is associated with impaired OXPHOS and ATP production in muscle mitochondria [14]. Such a compromised function of mitochondrial metabolism is also observed in ß-pancreatic cells of T2D patients [15], together with an increase of mitochondrial membrane potential (ΔΨm) and intracellular Ca^2+^ content [16]. In adipocytes of obese patients, a reduction of mitochondrial oxidative capacity (oxygen consumption rate) is observed [17] and is associated with a decrease of mitochondrial biogenesis [18]. During tumorigenesis, transformed cells undergo metabolic reprogramming allowing them to sustain the increased metabolic needs of the highly proliferation rate. This metabolic switch, characterized by a high rate of aerobic glycolysis (Warburg effect) allows the generation of glycolytic intermediates to support anabolic reactions leading to the biosynthesis of lipids, proteins and nucleotides. The activation of the pentose phosphate pathway also provides NADPH to augment antioxidant defense and biosynthetic processes [19]. An abundant literature describes preclinical assays in which strategies to target metabolic enzymes for cancer therapy have been tested (reviewed in [19]). For example, a reversion of metabolic reprogramming occurring during cell transformation (i.e., Warburg effect) leads to an attenuation of tumor phenotype (decreased cancer cell proliferation and reduced tumor growth) [20,21]. In clinical trials, some drugs have been tested, such as dichloroacetate (DCA), an inhibitor of pyruvate dehydrogenase (PDH) kinases, that re-activates the activity of PDH leading to decreased glycolysis to the benefit of oxidative phosphorylation [22,23]. Some mitochondria-targeting drugs have also been used to counteract mitochondrial dysfunction in other diseases. For example, metformin is used for decades as the first-line therapeutic choice for the treatment of T2D due to its effective glucose-lowering abilities [24]. Mitochondrial respiratory-chain complex I has been described as the primary target of metformin, with an inhibition resulting in decreased NADH oxidation, reduced proton gradient across the inner mitochondrial membrane, and decreased oxygen consumption rate (reviewed in [25]).

ROS have been identified as signaling molecules that promote crosstalks between mitochondria and other organelles. A low level of ROS promotes lifespan such as caloric restriction, exercise and intermittent fasting by triggering adaptative response (mitohormesis) [26], whereas increased levels of ROS induced by mitochondrial dysfunctions are involved in aging process and multiple disease development. Impaired homeostasis of mitochondrial metabolism and OXPHOS can cause an increase of oxidative stress (imbalance between ROS generation and antioxidant cellular defenses), leading to perturbation of cell signaling and molecular damages to lipids, proteins and DNA [27]. The ROS production is controlled by various factors including a dysfunction of electron transport through the MRC, disrupted ΔΨm, availability of oxygen and redox status [28]. The major source of ROS production is the MRC, mainly through the activity of complexes I and III. Deregulation of complex II, although less explored, can also lead to the production of ROS [29,30]. In particular, a significant increase in the concentration of succinate could promote a return of electrons from complex II to I and thus participate in the augmentation of ROS production under these conditions. The generation of ROS is increased by the blockade of the electron transport, typically when the F0–F1 ATPase is inhibited, and reversed by a dissipation of the electrochemical potential. The ROS production may be stimulated also under hypoxic conditions leading to the stabilization of hypoxia inducible factors (HIFs) and the subsequent activation of genes encoding proteins involved in an adaptive metabolic response. The role of ROS in aging is well described as a key modulator of metabolic diseases such as T2D and insulin resistance, neurodegenerative diseases, and cancer. In cancer cells, excessive ROS production promotes cancer initiation and progression in part through ROS-mediated mutations in mtDNA and defects in the mtDNA repair system. Thus, various tumor cells display a high rate of mutations in mtDNA and alterations of the mtDNA replication leading to mitochondrial dysfunction and in return, to the production of ROS, as a vicious cycle [31]. During ischemic stroke, sudden decreased blood flow due to the occluded vessels induces oxygen deprivation that causes brain damage. Upon reperfusion, the oxygen influx induces a high-level production of ROS in the ischemic brain tissue whereas antioxidants are consumed and detoxification pathways are inactivated. This cerebral ischemic cascade results in neuronal damages and neuron degeneration and is associated with inflammation processes [32,33]. Mitochondrial dysfunctions and ROS production are also involved in changes in epigenetic marks of mtDNA that might contribute to neurodegeneration in Parkinson and Alzheimer diseases, with impaired methylation of the D-loop region of mtDNA involved in replication [34].

Mitochondrial dynamics participate in multiple cellular processes such as cell cycle, differentiation, apoptosis and mitophagy, and appear to be essential for mitochondrial functions [35,36]. Thus, the morphology of mitochondria and the architecture of mitochondrial network are extremely flexible and their modifications play a central role for metabolic adaptation required to fulfill cell needs. Fusion and fission allow mitochondria to share membranes, metabolites and proteins. The fusion process is controlled by three “fusogenic” GTPases: Mitofusin 1 (MFN1) and Mitofusin 2 (MNF2) located at the outer mitochondrial membrane, and Optic Atrophy 1 (OPA1) located at the inner mitochondrial membrane. The fission event involves several proteins but the cytoplasmic GTPase Dynamin related protein 1 (DRP1) has been identified as a key player. The mitochondrial network is labile and its modifications are strongly linked to the metabolic state of cells, nutrient availability and energy needs. Mitochondrial fusion leads to large mitochondrial networks that regulate mitochondrial bioenergetics, providing advantage to cells under high energy demands. Gain/loss of function studies have shown that MFN2 overexpression causes an increase of oxidative capacities, TCA activity and ΔΨm. By contrast, MNF2 downregulation triggers a drop of oxidative capacities, cell respiration and TCA cycle activity that are compensated by an increase of glucose uptake and a switch toward glycolysis to produce ATP [37]. Mitochondrial defects are associated with various neurodevelopmental or neurodegenerative manifestations, underlying the importance of mitochondrial function in brain development and neuronal differentiation. Then, both in embryonic and adult neural stem cells (NSCs), mitochondria appear fractionated and the energy metabolism mostly glycolytic. Upon their commitment in the neuronal lineage, NSCs display mitochondrial biogenesis and elongation prior switching their metabolism toward an oxidative phenotype [38]. Mitochondrial morphology and dynamics are also impaired in some diseases such as autism spectrum, amyotrophic lateral sclerosis, Huntington’s and Alzheimer’s diseases, with increased fragmentation of mitochondria associated with oxidative stress [9]. In some cancer cells, high levels of mitochondrial fission activity are associated with high proliferation and invasiveness [39]. Deregulated mitochondrial dynamics are observed differentially, depending on the oncogenic pathway. Thus, the transformation of fibroblasts with Ras oncogene induces an overexpression of DRP1 and mitochondrial fragmentation in association with decreased mitochondrial respiration and ATP production [40]. In Burkitt lymphoma P493 cells, Myc oncogene loss induces the deregulation of expression of thousands genes of which several hundred are involved in mitochondrial function. Then, a number of genes encoding proteins involved in organelle shape, size and quality control has been identified as major targets of Myc [41]. DRP1 overexpression is observed in human breast carcinomas and cancer cells in lymph nodes, comparatively to that of non-metastatic carcinomas or adjacent normal tissue, suggesting that fragmentation is associated with cancer progression. Strengthening this hypothesis, mitochondrial fission is associated with cell spreading and lamellipodia formation that characterizes migration properties of invasive cancer cells [42].

## 3. Mitochondrial Dysfunction upon Exposure to Environmental Pollutants: Multiple Mechanisms of Toxicity

**Figure 2:**
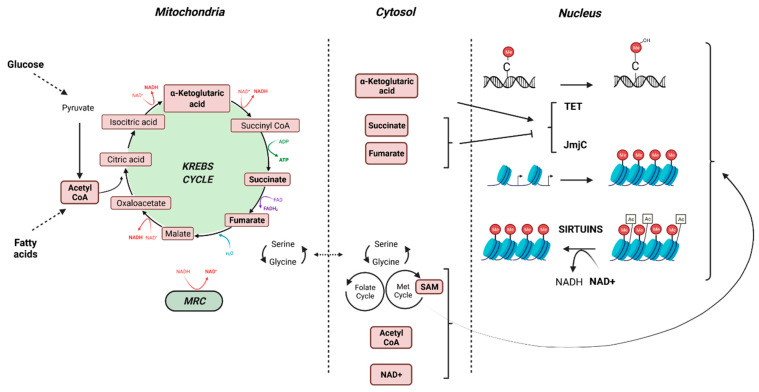
Crosstalk between the mitochondria and the epigenome (created with BioRender.com, accessed on 30 November 2021).

Human aging is characterized by an increase in the incidence of chronic diseases. Lifestyle risk factors such as diet, physical exercise, sedentary lifestyle, smoking or alcohol play a key role in the emergence and evolution of chronic diseases. Exposure to multiple environmental contaminants throughout life has a significant impact on health, at various degrees depending on the period of life to which one is exposed, the chronicity and the doses of exposure. Contaminants display various mechanisms of toxicity. They can sustainably modify gene expression, disrupt metabolic homeostasis, induce oxidative stress, lead to endocrine signaling dysfunction, or perturb epigenetic marks. As a central hub of all these mechanistic endpoints, a growing amount of data provided by academic scientific literature points to mitochondria as a key organelle targeted by environmental pollutants [43], opening new fields in mechanistic toxicology, largely unexplored by regulatory toxicology (Figure 2).

A number of environmental pollutants, including polycyclic aromatic hydrocarbons and pesticides have been identified as metabolic disrupting chemicals [44], and may cause off-target mitochondrial deregulation. Growing literature highlights dysfunction of the MRC upon toxicant exposure. Several herbicides, fungicides, insecticides and acaricides alter the activities of MRC complexes leading to decreased ATP levels, membrane depolarization and ROS production [45]. For example, pyrethroids induce a decreased ΔΨm and an increased permeability, and reduce the expression of cytochrome *c*, thus depressing the activity of cytochrome *c* oxidase in rat brain [46]. They may also induce a reduced activity of the complex I of the MRC associated with a nigral dopaminergic neurodegeneration and microglial activation, as observed in Parkinson’s disease [47]. An increasing corpus of literature also supports the role of persistent organic pollutants (POPs) as “mitotoxicants” involved in metabolic diseases such as obesity and T2D [48]. The usage of the persistent herbicide atrazine strongly overlaps with the obesity map, suggesting that heavy exposure to atrazine may be associated with the risk of developing this pathology [49]. Supporting this observation, chronic exposure to low concentrations of atrazine in rats induces abdominal obesity and insulin resistance through an alteration of mitochondrial function mediated by a blockade of complexes I and III of the MRC, resulting in decreased O_2_ consumption [49].

An alteration of mitochondrial dynamics also contributes to mitochondrial dysfunction in link with insulin resistance and T2D [50,51]. In the liver of cadmium-treated rats, mitochondrial fragmentation and mitochondrial dysfunction are observed, at least in part through a reduced expression of DRP1 and an enhancement of its recruitment into mitochondria. These effects are associated with an increase of ROS production, a drop of ΔΨm and a decrease of ATP production [52]. In rat hepatocytes, benzo(a)pyrene (B(a)P) induces a metabolic reprogramming sustaining survival signal, characterized by a decreased cell respiration rate with a drop in complex II activity and an increased lactate production [53]. These observations are also associated with an increased ROS production, a hyperpolarization of the mitochondrial membrane and an elongation of mitochondria suggesting a deregulation of mitochondrial dynamics [54,55].

Mitochondria crosstalks with epigenetic marks may be disturbed by environmental pollutants. Most metabolites and co-substrates involved in epigenetic patterning derived (directly or indirectly) from mitochondrial metabolism. Acetyl coenzyme A (acetyl-coA) is produced from pyruvate, the end-product of glycolysis, by PDH in the mitochondrial matrix. It also can be produced by fatty acid oxidation. Acetyl-coA enters the TCA cycle through its combination with oxaloacetate to form citrate that can either continue to run the Krebs cycle, or, if in excess, leave the Krebs cycle to participate in the lipogenesis in the cytoplasm. Acetyl-coA is also a substrate for the acetylation of proteins, including histones. Reduction of acetyl-coA levels through a limitation of glucose or lipids leads to decreased levels of histone acetylation and consequently augments DNA condensation [56]. The essential cofactors NAD^+^ and NADH are distributed both in cytosol and in mitochondria and the ratio NADH/NAD^+^ is closely related to nutritional state and mitochondrial function. The levels of NAD^+^ and NADH are critical for mitochondrial metabolism, as the Krebs cycle and MRC require NAD^+^ and NADH, respectively. Moreover, NAD^+^ is a substrate of sirtuin–histone deacetylases (SIRT), in which activation promotes histone deacetylation, recruitment of DNA binding proteins, condensation of chromatin and downregulation of gene expression [56]. S-adenosylmethionine (SAM) is produced in the cytosol from L-methionine and ATP. The generation of L-methionine from homocysteine depends on serine biosynthesis that also requires NAD^+^. Then, high levels of SAM are generated when glucose concentration/availability is low (fasting, starvation) and NADH/NAD^+^ ratio is reduced. Inhibition of OXPHOS promotes an increase of mitochondrial NADH/NAD^+^ ratio, leading to a diminution of serine biosynthesis and hence a reduced production of SAM [56]. SAM is used for post-translational modification of histones that can be mono-, di-, or trimethylated on lysine and arginine residues through the transfer of a methyl group by histone methyltransferases. SAM is also a substrate for DNA methylases to methylate cytosines of DNA, leading to reduced expression of the hypermethylated genes. Mitochondrial metabolism also provides substrates such as α-ketoglutarate, succinate and fumarate that modulate the activity of Jumonji-KDM histone demethylases and TET DNA demethylases. Thus subtle variations in the concentration of these metabolites may compromise epigenetic patterning with consequences on gene expression and thus perturbation of cellular function with consequences on the development of various diseases including cancer [57] (Figure 2).

Altogether, these mechanisms allow to consider the epigenetic effects of environmental chemicals partly as a consequence of mitotoxicity. Epigenetic modifications are increasingly mentioned to explain the impact of toxicants on health, in particular through the DOHaD (Developmental Origins of Health and Disease) concept. Thus, chronic diseases of adulthood may have, at least partially, an early origin, emphasizing fetal and early life development as crucial windows of vulnerability. Conclusive evidence from in vivo and in vitro studies show that exposure to DDE (Dichlorodiphenyldichloroethylene), hexachlorobenzene, tributyltin, bisphenol A or a mixture of organochlorines in early life may promote the emergence of obesity and metabolic syndrome in later life [58], in particular, but not only, in a context of high fat diet [59]. These obesogenic effects may be associated with epigenetic changes. For example, the increased body weight and adipogenesis observed upon exposure to the fungicide tributyltin during gestation is associated with an hypomethylation of the promoter of the adipogenic differentiation gene marker fatty acid-binding protein 4 (Fabp4) that target PPARγ [60]. The flame retardant 2,2’,4,4’-tetrabrominated diphenyl ether (BDE-47) also promotes adipocyte differentiation in the murine 3T3-L1 cells in association with a demethylation of three CpG sites in the PPARγ promoter, leading to its activation and the potential disruption of glucose homeostasis [61]. Epigenetic dysregulation is suspected to participate in bisphenol A toxicity in metabolic disorders, with aberrant DNA methylation, histone demethylation and deacetylation of the promoters of genes involved in carbohydrate-lipid homeostasis such as Pdx1, Gck, Cpt1, Srebf1 [62]. More importantly, epigenome modifications under exposure to environmental pollutants in early life are transmissible to later generations. For instance, bisphenol A and phthalates promote epigenetic transgenerational inheritance of adult obesity in further generations, with marked effect in F3 generation in link with epigenome changes [63]. Similarly, tributyltin exposure during the fetal period predisposes unexposed F4 male descendants to obesity under a moderate high-fat diet by altering chromatin organization as a result of global changes of DNA methylation [64]. Modulation of mtDNA methylation patterns also have been observed upon exposure to environmental toxicants such as airborne pollutants, flame retardants, maternal smoking and endocrine disruptors [65,66,67,68].

Although pharmacological manipulations have tried to counteract mitochondrial dysfunction in various diseases, to the best of our knowledge concerning contaminants, no clinical interventions have been tested to correct pollutant-induced mitochondrial dysfunction. Obviously, it could be expected that some defects disappear when the toxicants are removed, while others become permanent and transmissible, such as epigenetic marks.

## 4. AhR as a Crucial Player in Environmental Pollutant-Induced Mitochondrial Dysfunction

Upon pollutant exposure, xenobiotic receptors including AhR trigger the activation of detoxification enzymes involved in the biotransformation of the parent substances into metabolites to promote their elimination. AhR acts as an “exposome receptor”: it has been characterized initially as a receptor of polycyclic aromatic hydrocarbons or dioxins; new ligands have been discovered in food or microbiota but also produced by the body (endogenous ligands). Each ligand leads to an adaptive metabolic response which varies according to its nature. For example, TCDD (2,3,7,8-TetraChloroDibenzo-p-Dioxin) binds to the AhR which dimerizes in the nucleus with its partner, ARNT (AhR Nuclear Translocator), forming a transcription factor which transactivates xenobiotic metabolizing enzyme genes. Since 20 years, new stress responses have been characterized, including metabolic adaptations or in the case of long-term exposure, metabolic disruptions due to persistent AhR activation involving mitochondria dysfunction.

These alterations have been observed in a variety of tissues:

In the liver, activation of the AhR by TCDD leads to oxidative stress, and one of the mechanisms evoked is a disruption of mitochondrial metabolism with an hyperpolarization of its inner membrane and subsequently, a decrease in cellular respiration and ATP production [69]. The role of the AhR is essential in this process as AhR-knockout mice treated with 15 µg TCDD/kg whose livers are recovered and analyzed 1-week post-exposure, display a collapsed production (20%) of ROS. The role of family 1 cytochromes P450 (CYP1) that can also form ROS during catalysis, is suggested [70]. Nevertheless, the increase of ROS production in wildtype mice is also observed in Cyp1a1- or Cyp1a2-knockout mice suggesting that another pathways are involved including mitochondrial function [71]. Indeed, using the same models, another study found the same relationship after exposure to either TCDD or cigarette smoke in the pancreas [72].

An over-activation of complex I activity could be at the origin of these observations as suggested by in vitro experiments performed on mitochondria isolated from Hepa1c1c7 or Hepa-C12 liver cells treated for a few hours with TCDD [73]. Treatment of ovariectomized C57BL/6 female mice (to limit crosstalk effects between estrogens and TCDD [74]), with relatively high doses of TCDD, leads to an increase in hepatic mRNA levels of several proteins forming MRC complexes (with the exception of complex II). These regulatory effects at the level of transcripts, which could impact the levels of proteins and activities, are not the only proposed mechanism to link AhR with mitochondrial disruption. While the cytoplasmic-nuclear translocation is the main process described following the binding of ligands to the AhR, a localization of the receptor at the mitochondrial inner membrane has been identified [73]. Thus, a direct interaction between the AhR and the ATP51 subunit of the complex V (ATP synthase) has been observed, which has been linked to a hyperpolarization of the inner membrane consistently with what is described above [69]. This localization of the AhR to the inner membrane is thought to depend on a mitochondrial outer membrane translocase 20 (TOMM20). Other teams link the increase in ROS production in the liver of AhR-activated mice to a decrease in the activity of superoxide dismutase 2 (SOD2 or MnSOD) and therefore, to a decrease in ROS clearance. Two mechanisms have been described linking the AhR to the decreased SOD2 activity: (1) the interaction between the mitochondrial fraction of CYP1B1 (a direct AhR transcriptional target) and SOD2, and (2) the induction of TiPARP, a TCDD-induced poly(ADP-ribose) polymerase, which utilizes as a substrate, the coenzyme NAD^+^, subsequently leading to deactivation of the mitochondrial deacetylase Sirt3. This leads to an increased acetylation of SOD2, a post-translational modification associated with a decrease in its activity [75].

In terms of public health consequences, exogenous AhR ligands such as B(a)P could be responsible, via this induced mitochondrial dysfunction and overproduction of ROS, to the appearance of characteristic markers: (i) steatohepatitis (using an in vitro liver models) or (ii) pancreatitis (using mice) [72,76].

Besides the liver and pancreatic systems, AhR activation also increases the production of ROS at the mitochondrial level and induces apoptosis in cardiomyocytes in zebrafish embryos. This phenomenon, which is associated with malformations of the developing heart, is blocked by the AhR pharmacological antagonist CH-223191 [77]. In the absence of exogenous ligand, AhR can be activated by other stresses such as cardiac ischemia-reperfusion, which is characterized by a hypoxic phase related to the absence of blood flow in the heart followed by a high O_2_ resupply. In this context, similarly to what is observed in the liver, the AhR is partly translocated into the mitochondrial intermembrane space, activating the mitochondrial apoptosis pathway that finally leads to the death of myocardial cells [78].

Disruption of the ΔΨm can also be observed in epididymal spermatozoa upon treatment with TCDD, for example, of male C57BL/6 mice (0.1–50 µg/kg, 24 h). This effect (also measured in vitro) is not observed in AhR-knockout mice [79].

Observations performed in the lung complexify the role of AhR in these mitochondrial-dependent processes; indeed, at the pulmonary level, AhR over-expression in fibroblasts prevents apoptosis caused by cigarette smoke. AhR regulates fibroblast proliferation whereas its absence leads to cleavage of both PARP and caspase-3 as well as mitochondrial dysfunction with release of cytochrome *c*; this is also true in lung epithelial cells whose sensitivity to cigarette smoke-induced apoptosis increases in the absence of AhR [80]. While the presence of the AhR appears to be protective, it is important to note that AhR ligands in this tissue also increase the production of ROS. This contributes to the stimulation of the ERK and cAMP/CREB pathways, which induces the mRNA and protein expression of MUC5AC, a constituent of pulmonary and bronchial mucins; the overproduction of mucus may be associated with a higher risk of respiratory diseases [81].

In summary, most observations suggest that activation of the AhR by exogenous ligands leads to a disruption of mitochondrial functions, even though some studies also describe a protective effect of the receptor. As suggested by the review of Brinkmann et coll. [82], the effects of the AhR on mitochondrial functions are likely to be tissue-, age-, and even gender-dependent. As suggested by additional studies, the functions of the receptor should also be considered upon the timing of its stimulation (chronic vs. acute) and the nature of the ligands, which could be metabolized and inactivated; for example, while TCDD and most dioxins are persistent pollutants, which are not transformed by CYP1 enzymes, most natural and microbial products are rapidly eliminated.

Most studies report that chronic activation of the AhR is often associated with the stimulation of apoptotic processes. For example, the concentration of AhR ligands in the serum of individuals is correlated with clinical markers of metabolic syndrome (glucose intolerance), obesity (body mass index), elevated blood pressure or triglyceride levels. If myoblasts are incubated with these human sera, the ability of mitochondria to generate ATP decreases with the capacity of the samples to stimulate the AhR. On the contrary, in case of acute activation, protective mechanisms are often described: B(a)P stimulates adaptive mitochondrial responses such as autophagy and mitophagy. The activation of these processes, for example in HaCaT cells (human keratinocytes), is dependent on the presence of AhR and its target gene, CYP1B1; autophagy is also dependent on Beclin-1 via the mTOR/AMPK pathway. B(a)P also decreases (i) the ∆Ψm of these cells, leading to a decrease in ATP levels and inhibition of the rate of O_2_ consumption and (ii) the activity of a superoxide dismutase (MnSOD) probably by increasing the levels of mitochondrial CYP1B1 which interacts physically with this MnSOD. These processes cause mitophagy which temporarily protects the cell from potential B(a)P-induced apoptosis. Moreover, if HaCaT cells are deficient in the establishment of autophagy, B(a)P exhibited higher cytotoxicity [83].

Similarly, low concentrations of fine particles (PM2.5) of Parisian origin, in particular their fraction containing aromatic hydrocarbons, display anti-apoptotic effects on different models of bronchial epithelial cells (cell lines, primary cultures). It is possible that, in this specific context, the low concentrations of PM contribute to a low exposure of PAHs which are rapidly metabolized [84]. Furthermore, in the absence of exogenous ligand, the AhR could play a major role in the protection of melanocytes against oxidative damage, inducing mitochondrial biogenesis [85].

**Figure 3:**
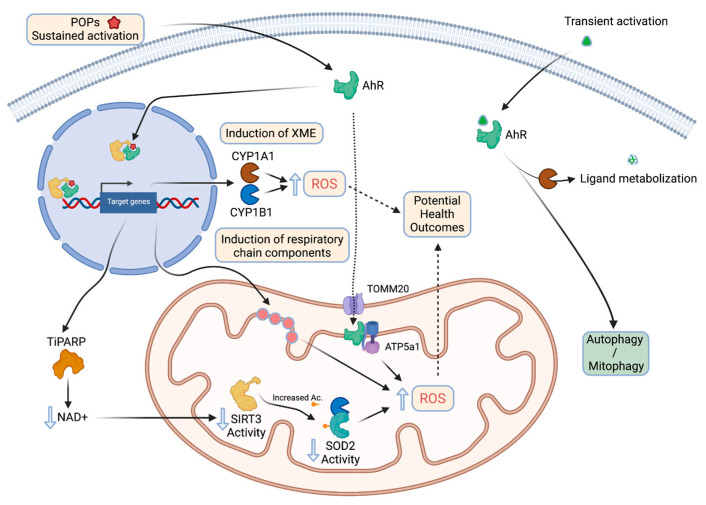
The relationship between AhR chronic vs acute activation by its ligands and mitochondrial functions (created with BioRender.com, accessed on 30 November 2021).

In conclusion, the effects of endogenous ligands or low doses-exogenous ligands (as opposed to persistent exogenous ligands [72,86]) would have cell-protective effects as they only lead to transient AhR activation and possibly mitogenesis promoting effects (Figure 3) [87].

Besides the AhR, other xenobiotic receptors such as CAR or PXR regulate or target the mitochondrial functions but the number of studies is much lower than the ones involving the AhR: a recent work shows that PXR (pregnane X receptor) targets an enzyme, the aldo-keto reductase family 1, member B7 (AKR1B7), which significantly improves mitochondrial function in acute kidney injury (AKI). This is confirmed using an ischemia/reperfusion model leading to acute kidney injury [88]. Given the wide variety of pharmacological and toxicological ligands that can target this receptor, these results are of primary importance. Regarding CAR (constitutive androstane receptor), its activation by overexpression results in a stimulation of the mitochondrial energy metabolism, but also a decrease in the production of mitochondrial superoxide anion levels [89].

Finally, diverse receptors which have been historically identified as receptors of endogenous ligands could also be targeted by environmental chemicals and subsequently disrupt the mitochondrial functions. For example, mitochondria are a target of estrogens. One of their receptors, ERβ, is expressed at the mitochondrial level and regulates, in part, mitochondrial energy activity and the mitochondria-dependent apoptotic pathway. The activation of ERβ by xenoestrogens could therefore alter positively (agonists) or negatively (antagonists) these processes [90].

## 5. Concluding Remarks Related to Mitotoxicity and Risk Assessment

The systemic toxicity of environmental contaminants is explored using animal models (mainly rodents) submitted to acute and chronic exposure of pollutants. These studies aim to determine the effect of a substance on clinical, physiological and biochemical parameters at the level of the whole organism and to identify targeted organs. They explore the impact of contaminants on genotoxicity, mutagenicity, carcinogenesis, reproduction, development and neurological function. At the risk level, they are also used to derive toxicity reference values such as ADI (Acceptable Daily Intake), ARfD (Acute Reference Dose) and AOEL (Acceptable Operator Exposure Level). Besides, the perturbation of cellular functions under toxicants exposure is investigated by mechanistic toxicology using a battery of assays mainly focused on genotoxicity, cytotoxicity and cell proliferation. However, new insights indicate that environment-induced mitochondrial dysfunction lead to metabolic reprogramming, oxidative stress, alteration of mitochondrial dynamics and deregulation of epigenetic marks (Table 1).

Mitochondrial dysfunction appears to have a central role in the triggering and the progression of numerous human diseases, highlighting a strong susceptibility of mitochondria to environmental pollutants. Despite deregulation of mitochondrial function is suspected to play a key role in numerous environment-related chronic diseases, mitotoxicity is still largely unexplored in regulatory toxicology. Growing evidence emphasizes close interactions between mitochondrial metabolism and the epigenome, revealing that subtle variations of mitochondrial metabolite content may disturb epigenetic marks. For example, we highlighted in this review the specific effects of TCDD on the acetylation of SOD2 due to the decrease of NAD^+^, a substrate of SIRT3. We could hypothesize that this decrease in NAD^+^ probably impacts the activity of other sirtuins (SIRT) leading to the alteration of epigenetic marks, which results in the disruption of processes related to functions of the mitochondria. Moreover, other xenobiotic receptors such as CAR modulates the metabolism of carbohydrates and lipids which could also impact the intracellular levels of coenzymes.

The modifications of epigenome due to environmental insults involving mitochondria dysfunctions could also be reflected on long term: transgenerational studies have shown that fetal exposure to pollutants may have consequences on adult heath of further generations underlying windows of vulnerability during development. Altogether, the involvement of mitochondrial dysfunctions in environment-related pathologies opens a large field of investigation that requires the development of reliable and robust studies for an in-depth understanding of the mechanisms involved.

## Figures and Tables

**Table 1:** Table summarizing the key features of the mitochondria (1st column) and the potential consequences of exposure to xenobiotics (2nd column).

Key Features of the Mitochondria	Consequences Regarding the Exposure to Xenobiotics
Mitochondria are complex organelles whose functions are governed by multiple and diverse proteins	As a result, a wide variety of xenobiotics can target the mitochondria such as polycyclic aromatic hydrocarbons, pesticides (including rotenone, pyrethroids, atrazine), metals (cadmium).
Mitochondria are involved in the production of ATP which is coupled with the activity of the respiratory chain	Both processes (ATP production and respiration) are governed by complexes of the inner membrane which are targeted by natural and foreign compounds. Disruption of the respiratory chain can lead to the misuse of O_2_ and production of reactive oxygen species. It can also promote the Warburg Effect.
Mitochondria regulate multiple cellular processes including cell proliferation, differentiation, apoptosis and mitophagy	Exposure to xenobiotics targeting the mitochondria can lead to disruption of such processes, leading to various outcomes (increased apoptosis and neurodegeneration; increased survival in cancer).
Mitochondria crosstalk with epigenetics by regulating the levels of NAD^+,^ S-adenosylmethionine, α-ketoglutarate, succinate and fumarate which impact epigenetic marks.	Several pollutants impact epigenetic marks through the deregulation of mitochondria-related metabolites and/or co-factors production, such as DDE, hexachlorobenzene, tributyltin, bisphenol A, phthalates or organochlorines
Mitochondria contain transcriptional factors that regulate some of the functions mentioned earlier.	Binding of ligands to such transcriptional factors may lead to relocalization of these proteins to mitochondria and alteration of their properties (i.e., AhR and the Seveso dioxin) due for example to increased ROS production
Some pollutants are non-persistent while other are persistent (with a long-half life in the body)	The nature of each xenobiotic is crucial to determine their long-term effects on the mitochondria: endogenous ligands or low doses-exogenous ligands can then be opposed to persistent exogenous ligands regarding the potential health outcomes they would induce
